# Disentangling drivers of the abundance of coral reef fishes in the Western Indian Ocean

**DOI:** 10.1002/ece3.5044

**Published:** 2019-03-21

**Authors:** Melita A. Samoilys, Andrew Halford, Kennedy Osuka

**Affiliations:** ^1^ CORDIO East Africa Mombasa Kenya; ^2^ Department of Zoology University of Oxford Oxford UK; ^3^ Pacific Community (SPC) Noumea Cedex New Caledonia

**Keywords:** biogeography, biomass, climate, coral reef, fish, geomorphology

## Abstract

**Aim:**

Understanding the drivers of the structure of coral reef fish assemblages is vital for their future conservation. Quantifying the separate roles of natural drivers from the increasing influence of anthropogenic factors, such as fishing and climate change, is a key component of this understanding. It follows that the intrinsic role of historical biogeographical and geomorphological factors must be accounted for when trying to understand the effects of contemporary disturbances such as fishing.

**Location:**

Comoros, Madagascar, Mozambique and Tanzania, Western Indian Ocean (WIO).

**Methods:**

We modeled patterns in the density and biomass of an assemblage of reef‐associated fish species from 11 families, and their association with 16 biophysical variables.

**Results:**

Canonical analysis of principal coordinates revealed strong country affiliations of reef fish assemblages and distance‐based linear modeling confirmed geographic location and reef geomorphology were the most significant correlates, explaining 32% of the observed variation in fish assemblage structure. Another 6%–8% of variation was explained by productivity gradients (chl_*a*), and reef exposure or slope. Where spatial effects were not significant between mainland continental locations, fishing effects became evident explaining 6% of the variation in data. No correlation with live coral was detected. Only 37 species, predominantly lower trophic level taxa, were significant in explaining differences in assemblages between sites.

**Main Conclusions:**

Spatial and geomorphological histories remain a major influence on the structure of reef fish assemblages in the WIO. Reef geomorphology was closely linked to standing biomass, with “ocean‐exposed” fringing reefs supporting high average biomass of ~1,000 kg/ha, while “lagoon‐exposed fringing” reefs and “inner seas patch complex” reefs yielded substantially less at ~500kg/ha. Further, the results indicate the influence of benthic communities on fish assemblages is scale dependent. Such insights will be pivotal for managers seeking to balance long‐term sustainability of artisanal reef fisheries with conservation of coral reef systems.

## 1 INTRODUCTION

Biodiversity is declining globally as a result of direct human impacts including overexploitation of natural resources which in turn threatens ecosystem functioning (Butchart et al., 2010; Mora et al., 2011). Coral reefs are one of the most biodiverse ecosystems on the planet and of great importance for livelihoods and economies (Hoegh‐Guldberg et al., 2007; Moberg & Folke, 1999). These issues come together in the Western Indian Ocean (WIO) where coral reefs are associated with developing countries with artisanal fisheries of high socioeconomic value for poor coastal communities, though in many areas fisheries management measures are inadequate (Samoilys, Osuka, Maina, & Obura, 2017; Walmsley, Purvis, & Ninnes, 2006; Wells, Samoilys, Makoloweka, & Kalombo, 2010).

Biodiversity loss and human impacts on coral reefs are further exacerbated by climate change (Bellwood, Hughes, Folke, & Nyström, 2004; Hughes et al., 2007). Coral reefs are extremely vulnerable to rising sea surface temperatures resulting in mass coral bleaching (Ateweberhan, McClanahan, Graham, & Sheppard, 2011) and to ocean acidification, now driving some reefs into a state of net erosion (Hoegh‐Guldberg et al., 2007). The concomitant impact of coral bleaching and mortality on reef fishes has been well studied (Graham et al., 2011, 2007; Wilson et al., 2008). In addition, significant declines in reef fish biomass due to fishing in the Indo‐Pacific have been reported (Friedlander & DeMartini, 2002; McClanahan, Maina, Graham, & Jones, 2016; Sandin, Smith, et al., 2008). However, teasing apart natural drivers, climate change impacts and fishing effects on the structure of reef fish assemblages have been less clearly examined.

In this study, we sought to assess the relative impacts of natural versus anthropogenic factors that are affecting reef fish assemblages in the WIO. We structured our approach through two relatively well‐established hypotheses. The first was that fish assemblages will vary naturally in relation to a number of larger scale abiotic factors such as biogeography, reef structure, and oceanic nutrient levels, all either previously recognized or likely to have some influence in structuring coral reef communities (Heenan, Hoey, Williams, & Williams, 2016; Pinca et al., 2012; Taylor, Lindfield, & Choat, 2014). But at the smaller scale of reef habitat, the cover of hermatypic corals, algae, rubble, and rugosity also plays a significant role in structuring fish assemblages (Chabanet, Ralambondrainy, Amanieu, Faure, & Galzin, 1997; Halford, Cheal, Ryan, & Williams, 2004; Samoilys, Roche, Koldewey, & Turner, 2018). Our second hypothesis was that the abundance and biomass of coral reef fishes will vary in relation to protective management and fishing, which has been widely demonstrated in studies across the Indo‐Pacific (D'agata et al., 2014; DeMartini, Friedlander, Sandin, & Sala, 2008; Edgar et al., 2014; McClanahan, Graham, Calnan, & MacNeil, 2007). We maximized the range of depths of the surveys to be able to characterize the fish assemblage at each reef (Wedding & Friedlander, 2008) rather than select a small depth range to minimize data variance. Anthropogenic stressors, both positive (management) and negative (extraction through fishing), were represented by human population density, fishing pressure, and an index of management protection.

One of our primary objectives was to clarify significant drivers of fish assemblage structure while also paying due regard to the role of spatial autocorrelation in the misinterpretation of multiple regression models (Hawkins, 2012; Legendre, 1993). Spatial autocorrelation is a well‐known manifestation of community data which, if not explicitly accounted for in models, will inflate the significance of other terms in the model (Peres‐Neto & Legendre, 2010). The Western Indian Ocean (WIO) biogeographic region (Spalding et al., 2007) represents a region of highly variable coral reefs (Sheppard, 2000), within an oceanic context of the South Equatorial Current and the East African Coastal Current (Schott, Xie, & McCreary, 2009). This provided an ideal study area for enabling the collation of 16 explanatory variables to explore drivers of the structure of coral reef fish assemblages.

## 2 METHODS

### 2.1 Sites

**Figure 1:**
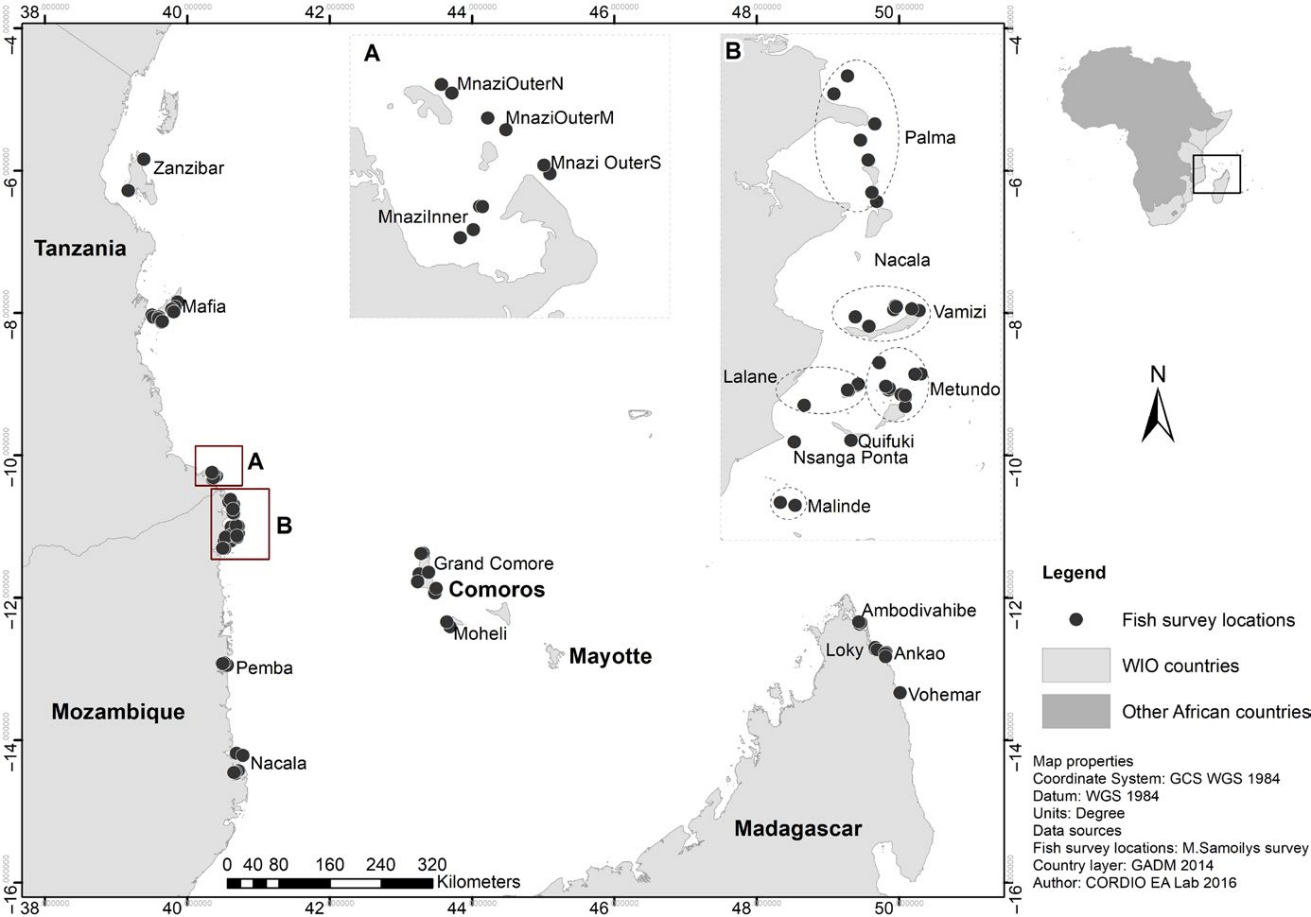
Map of the Western Indian Ocean (WIO) showing locations of underwater visual census sites in the four countries

Reef fish population abundance and benthic cover were measured at 53 coral reef sites across Tanzania, Mozambique, Comoros, and Madagascar (Figure 1), during 2009–2011, with some additional sites from Mozambique in 2014–2015. Site locations were selected to cover a range of geomorphologies and reef types, but primarily selected the deeper forereefs to maximize fish species richness and abundance (Table 1). Due to the extent of coral reefs in these countries, the study sites are not representative of each country as a whole. Reef geomorphology was categorized on a 3‐level hierarchical typology of coral reef types defined for the WIO based on 6 geological, 7 geomorphological, and 6 reef types (Table 1).

**Table 1:** Survey sites (*n* = 53) and their reef geomorphology (seven categories) and reef type (six categories, after Andréfouët, Chagnaud, & Kranenburg, 2009) OS=Outer Shelf

Country	Location	Geology (6)	Reef geomorphology (7)	Reef type (6)	No. sites
Comoros	Grande Comore	Oceanic Island	Ocean‐exposed fringing reef (24)	Forereef (28)	6
	Moheli	Oceanic Island	Inner seas‐exposed fringing reef (6)	Forereef	1
				Deep terrace (6)	1
Madagascar	Ambodivahibe	Continental Fringing Reef	Ocean‐exposed fringing reef	Forereef	1
				Shallow terrace (10)	2
	Ankao	Bank	Bank barrier (1)	Forereef	1
			Bank lagoon (2)	Shallow lagoonal terrace (6)	2
	Loky	Continental Fringing Reef	Ocean‐exposed fringing reef	Forereef	1
		Continental OS Barrier	Coastal barrier reef complex (9)	Diffuse fringing reef (3)	1
	Vohemar	Continental OS Barrier		Forereef	1
Mozambique	Lalane	Continental Patch Complex	Inner seas patch reef complex (7)	Shallow lagoonal terrace	1
				Shallow terrace	1
	Malinde	Continental Patch Complex	Inner seas patch reef complex	Shallow terrace	1
	Metundo	Continental Island	Coastal barrier reef complex	Deep terrace	2
				Reef flat (1)	1
				Shallow terrace	1
			Ocean‐exposed fringing reef	Forereef	1
	Nacala	Continental Fringing Reef	Inner seas‐exposed fringing reef	Diffuse fringing reef	1
			Inner seas‐exposed fringing reef	Forereef	2
			Lagoon‐exposed fringing reef (4)	Diffuse fringing reef	1
			Ocean‐exposed fringing reef	Deep terrace	1
	Nsanga Ponta	Continental Patch Complex	Inner seas patch reef complex	Shallow lagoonal terrace	1
	Palma	Continental Fringing Reef	Ocean‐exposed fringing reef	Forereef	2
		Continental Island	Coastal barrier reef complex	Forereef	1
			Coastal barrier reef complex	Shallow lagoon terrace	1
	Quifuki	Continental Island	Ocean‐exposed fringing reef	Forereef	1
	Vamizi	Continental Island	Coastal barrier reef complex	Deep terrace	1
			Ocean‐exposed fringing reef	Deep terrace	1
			Ocean‐exposed fringing reef	Forereef	1
Tanzania	Mafia	Continental Fringing Reef	Lagoon‐exposed fringing reef	Shallow lagoonal terrace	1
			Ocean‐exposed fringing reef	Forereef	3
		Continental Patch Complex	Inner seas patch reef complex	Shallow terrace	3
	Mnazi Bay	Continental Fringing Reef	Ocean‐exposed fringing reef	Forereef	3
		Continental Fringing Reef	Lagoon‐exposed fringing reef	Shallow terrace	2
	Zanzibar	Continental Island	Inner seas‐exposed fringing reef	Forereef	1
			Ocean‐exposed fringing reef	Forereef	1

Numbers in parentheses sum total number of sites per geomorphology type and reef type across all countries, illustrating most sites were forereefs.

### 2.2 Explanatory variables

Environmental variables that may influence patterns in reef fish assemblages were measured at each site and included in situ estimates of exposure to waves and trade winds, minimum and maximum depth, rugosity, and reef slope (Table 2). Other variables defined per site included chlorophyll_*a*, as a proxy for nutrient levels in the water, obtained from the ocean color CCI web GIS portal. Chl_*a* readings for each site were calculated from monthly averages between 1997 and 2013 from three pixels closest to each site. Two indices for anthropogenic variables were also defined per site: (a) human population density adjacent to the survey sites (sensu Taylor et al., 2014) and (b) an overfishing and destructive fishing threat measure (adapted from Burke, Reytar, Spalding, & Perry, 2011). Gridded human population data sourced at 4‐km^2^ pixel resolution (CIESEN, 2015; Doxsey‐Whitfield et al., 2015) were converted to a vector point layer using ArcGIS 10.3 and then projected onto geo‐referenced survey sites and the shortest distance between the two vector points taken to assign human population value per site. The fishing pressure threat index was derived from the World Resources Institute's evaluations of coastal population density and extent of fishing areas, with adjustments for increased fishing demand due to proximity to large populations and market centers. This adjustment involved all reef sites that were within 200 km of ≥500,000 people but were initially rated as low threat based on local population size. Such sites were reclassified to medium threat (sensu Burke et al., 2011). The threats ranged from low (0) to high (1,000). Areas where destructive fishing occurs (with explosives or poisons, MS pers. obs) were added. A protection index was assigned for each site based on the effectiveness of management determined from the literature, personal knowledge, and communicating with managers (Supporting Information Table S3).

**Table 2:** Final list of driver variables tested for influence on fish species’ population density and biomass

Driver variable	Description and source of data	Rationale and hypotheses	References
Geographic location	Latitude and longitude of reef site	Geography well‐known driver of reef fish assemblages in the I‐P	Mora (2015) and Jouffrey et al. (2014)
Reef geomorphology	Seven categories based on Level 3 in hierarchical description of reef types: Andréfouët et al. (2009)	Geomorphology known to drive parrotfish populations in the Pacific	Taylor et al. (2014) and Heenan et al. (2016)
Chlorophyll_a	Proxy for nutrient levels in seawater: ocean color CCI web GIS portal	Known to drive fish biomass in the Pacific	Williams et al. (2015)
Human population density	Global population density overlaid on survey sites to assign population density/16 km^2^: CIESEN (2015)	Human population density is a well‐tested proxy for fishing pressure and threats on coral reefs	Taylor et al. (2014) and Heenan et al. (2016)
Fishing pressure threat	Fishing threat index derived from World Resources Institute: Burke et al. (2011)	Fishing has a significant impact on reef fish population densities, biomass, and community structure	McClanahan et al. (2016) and Friedlander and DeMartini (2002)
Protection index	1–6 category index estimated for each survey site based on local knowledge	Protective management is a well‐known driver of healthy reef fish populations	McClanahan, Ateweberhan, Muhando, Maina, and Mohammed (2007) and Edgar et al. (2014)
Exposure to ocean waves	1–5 category index estimated for each survey site based on in situ observation and Google Earth	Lower fish abundance associated with high levels of wave exposure	Friedlander et al. (2003)
Reef slope	Visual estimate of slope in degrees following Sandin, Smith, et al. (2008)	Reef fish population abundances are known to vary with the degree of reef slope	Wedding and Friedlander (2008)
Depth	Two measures of depth at site were tested: minimum and depth range	Reef fish population abundance is known to vary with depth	Wedding and Friedlander (2008)
Rugosity	Visual estimate on site	High rugosity associated with higher biomass of reef fishes	Samoilys et al. (2018) and Heenan et al. (2016)
Reef benthos	% cover of five key benthic types measured in situ: hard coral, fleshy macroalgae, turf algae, CCA, and rubble, following Sandin, Smith, et al. (2008)	Reef benthos known to drive fish populations in multiple relationships	Chabanet et al. (1997) and Samoilys et al. (2018)

Note

See Supporting Information Tables S2 and S3 for further details of variables. References are far from exhaustive and are preferentially selected from Indian Ocean studies where available.

### 2.3 Benthic surveys

Benthic surveys estimated % cover of live hard coral, three types of algae and rubble (Supporting Information Table S2) using 25 × 1 m transects (sensu Sandin, Smith, et al., 2008), with generally two transects per site, at 47 of the 53 sites.

### 2.4 Fish surveys

The density and size classes of all reef fish species from 11 families and 12 trophic groups (Table 3; sensu Green & Bellwood, 2009) were counted using SCUBA‐based underwater visual census (Samoilys & Carlos, 2000) along 50 × 5 m transects (~*n* = 5 transects per reef site) on two dives. Occasionally, only three replicate transects were collected when only one dive was possible. All individual fish were identified to species level, with the exception of the Balistidae which were categorized as benthic or planktivorous; the Pomacanthidae which were recorded as invertivores (*Pomacanthus* spp. + *Pygoplites diacanthus *and *Apolemichthys* spp.) or grazer‐detritivores *(Centropyge* spp.) (Table 3). Trophic category of some parrotfish and surgeonfish changed with size reflecting ontogenetic shifts in diet (Table 3). Small individuals of parrotfishes (~5–10 cm TL) could not always be identified to species, so were recorded as *Scarus* spp. The size of all species >5 cm TL was estimated, and their biomass was calculated based on published length–weight relationships following procedures presented in Samoilys et al. (2018).

**Table 3:** Eleven families surveyed for abundance and biomass and their trophic group and functional characteristics

Functional group	Notes on feeding habits and selection of species	Group/family	English name or species
Piscivores	Top‐level predators, exert top‐down control on lower trophic levels of fish, are vulnerable to overfishing and therefore are good indicators of the level of fishing on a reef.	Serranidae Lutjanidae	All groupers *Aprion viriscens* *Lutjanus bohar*
Omnivores (omnivorous carnivores)	Second‐level predators with highly mixed diets including small fish, invertebrates, and dead animals. Their abundance is a good indicator of fishing pressure	Haemulidae Lethrinidae Lutjanidae	All sweetlip All emperor All snapper except *Aprion viriscens & Lutjanus bohar*
Corallivores	Obligate and facultative corallivores are a secondary indicator of coral community health.	Chaetodontidae	Eight Butterflyfish: *C. bennetti, C. lineolatus, C. melannotus, C. meyeri, C. ornatissimus, C. trifascialis, C. trifasciatus, C. zanzibarensis*
Invertivores	Feed on coral competitors such as soft corals and sponges, and their abundance may be a secondary indicator of stability of these groups and of a phase shift. Also prey on small invertebrates in the benthos.	Pomacanthidae	Angelfish. All species except *Centropyge* spp. which are grazer‐detritivores
		Balistidae	Benthic triggerfish (e.g., *Sufflamen* spp.)
		Chaetodontidae	Noncorallivore Butterflyfish: all other Chaeotdontids except *H. zoster *and *H. diphreutes* which are planktivores
Planktivores	Resident on reefs but feed in the water column. Their presence/absence may be related to water column conditions, suitable habitat for shelter or reef features such as passes.	Chaetodontidae	*Hemitaurichthys zoster, Heniochus *spp.
		Balistidae	Triggerfish in the water column eg. *Melichthys* spp., *Odonus niger*
		Acanthuridae	*A. mata, A. nubilus, A. thompsoni, Paracanthurus hepatus* All large *Naso* (>20 cm TL, 16 cm for *N. hexacanthus*), except Browsers (*N. unicornis *etc*)*
		Caesionidae	All Fusiliers
Detritivores	Feed on organic matter including diatoms in sediment and reef surfaces, high abundances poorly understood	Acanthuridae	*Ctenochaetus spp*.
Grazer‐detritivores	Feed on algal turf and sediment to extract detritus, microbes and diatoms; may limit growth of macroalgae	Acanthuridae	*A. blochii, A. dussumieri, A. leucocheilus, A. nigricauda, A. xanthopterus, A. tennenti*
		Pomacanthidae	*Centropyge *spp.
Herbivores	Feed on endolithic and epilithic algae, substratum, and macroalgae. Exert control on coral‐algal dynamics, implicated in determining phase shifts from coral to algal dominance, for example, in response to mass coral mortality		
Large excavators	Take few, large, deep bites, and remove calcareous substratum; play a large role in bioerosion	Scarinae	*Chlorurus spp. >35 cm, for example,* *C. strongylocephalos* *Cetoscarus ocellatus {Bolbometapon muricatum}*
Small excavators	Remove algae and substrate; play a smaller role in bioerosion	Scarinae	*Chlorurus spp. <36 cm*
Scrapers	Remove algae, sediment, and detritus by closely cropping or scraping the substrate		*Scarus spp., Hipposcarus spp*.
Browsers	Feed on large macroalgae	Scarinae	*Calotomus* spp. *Leptoscarus* spp.
		Acanthuridae	*Naso elegans, N. tuberosus, N. unicornis*, other *Naso spp*. <21 cm (<16 cm for *N. hecacanthus*)
Grazers	Graze epilithic algal turfs, including red algae; likely to limit growth of macroalgae	Acanthuridae	*Zebrasoma* spp. *A. nigrofuscus, *other *Acanthurus spp. for example,* *A. lineatus*
		Siganidae	*Siganus *spp.

Note

All taxa were recorded to species level (not all species are listed here). Those split by body size are species that change diet with size. Trophic categories and feeding information based on Choat and Clements (1998), Choat, Clements and, Robbins (2002), Samoilys and Carlos (2000), Green and Bellwood (2009) and Clements, German, Piche, Tribollet, and Choat (2016).

### 2.5 Data compilation

The complete fish dataset consisted of density and biomass for 156 species across 53 sites. Preliminary explorations identified two extreme outlier sites (Fernau Vloso & Lalane, Mozambique), which were therefore removed. To further reduce variance, any species occurring <3 times across all sites were removed. The final dataset for analyses consisted of 45 sites (Comoros—8, Madagascar—9, Mozambique—16, and Tanzania—12) for 123 coral reef‐associated fish species/taxa (see Supporting Information: Table S1).

The 16 potential explanatory abiotic and biotic variables (Table 2) were assessed for skewness and an appropriate transformation applied where necessary, followed by testing for collinearity through the use of variance inflation factors (VIF, Supporting Information Appendix S1). The presence of spatial autocorrelation was tested at two scales: across all locations and across locations within the eastern African mainland countries (Mozambique and Tanzania). See Supporting Information Appendix S1 for further details.

### 2.6 Analyses

Spatial patterns of the fish assemblages across sites were first examined through unconstrained clustering of Bray–Curtis similarities calculated on square‐root‐transformed abundance data using Ward's clustering algorithm. An IndVal analysis (De Caceres & Legendre, 2009) was performed to identify which species were significant delineators of the observed groupings and the distribution of species was displayed via a heat map. Further examination of spatial patterns was performed using canonical analysis of principal coordinates (CAP), to maximize differences between groups, using square‐root‐transformed abundance and log (*x* + 1) transformed biomass data. One‐way PERMANOVAs confirmed the significance of country differences for both abundance and biomass at (P(Perm) < 0.001). Sites were therefore coded by country a priori for the CAP. We identified which fish species were most influential in describing differences between countries by superimposing vectors representing Pearson correlations (|>0.45|) of individual species with the CAP axes (Anderson, Gorley, & Clarke, 2008). A complementary SIMPER analysis (Clarke & Gorley, 2015) was run to provide more quantitative information on species that were most influential in describing differences between countries.

Distance‐based linear models (DistLM) were run to investigate potential drivers of the observed reef fish assemblage patterns (sensu Legendre & Anderson, 1999), incorporating the 16 explanatory variables. Marginal tests were done on all variables to investigate the range of variation that could be explained. The BEST procedure was used for building the models with the best models chosen through AIC_c_ and BIC selection criteria (see Supporting Information Appendix S1). All analyses were performed using the PRIMER V7 software (Clarke & Gorley, 2015) with PERMANOVA add‐on (Anderson et al., 2008).

## 3 RESULTS

### 3.1 Spatial patterns in fish assemblages

**Figure 2:**
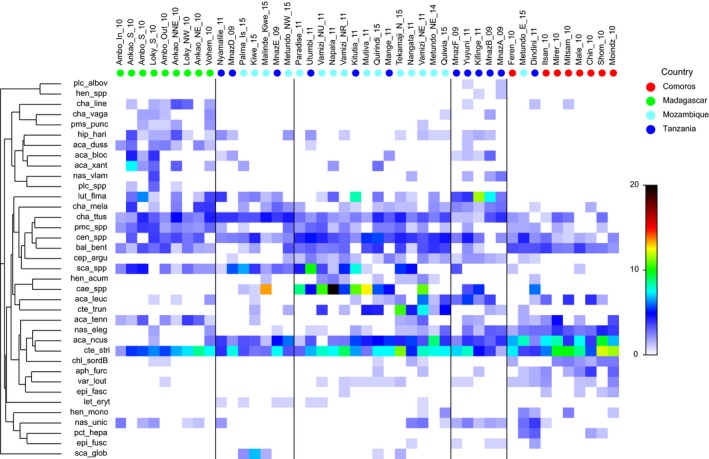
Heat map illustrating the spatial distribution and abundance (square‐root‐transformed) of the 37 species/taxa found to be significant indicators of the five cluster groupings from a total species list of 123 species. Significance derived from *IndVal* analysis (Dufrene & Legendre 1997). The 3rd and 4th groups illustrate the most diverse assemblages, while species that are ubiquitous spanning most sites are illustrated in horizontal bands

The cluster analysis on species density data identified five groups (similarity <0.7) and illustrated that sites in Comoros and Madagascar strongly separated from those in the adjacent mainland countries of Mozambique and Tanzania (Supporting Information Figure S1). Sites in Mozambique and Tanzania were more similar. The heat map (Figure 2) illustrates the 37 species that were significant indicators of the five groupings derived from the cluster analysis. The heat map also illustrates a core group of nine ubiquitous species consisting of three surgeonfish*, Acanthurus nigrofuscus, Ctenochaetus striatus*, and *Naso elegans, *one snapper*, Lutjanus fulviflamma, *two butterflyfish*, Chaetodon guttatissimus *and *C. melannotus, *and two angelfish genera (species lumped): *Centropyge *spp. and *Pomacanthus* spp.; and invertivorous balistids. While the first three species of this group were highly abundant across several locations, densities of the other species varied considerably (Figure 2). For example, *Lutjanus fulviflamma *was much more abundant in the mainland countries with the highest densities recorded in Mafia Island, Tanzania.

The heat map also illustrates those sites where species diversity was highest, with the widest spread of species in the third and fourth columns (Figure 2). These included sites from Mafia Island and Mnazi Bay, Tanzania, and sites in Palma and Vamizi and Metundo Islands in Mozambique, all largely offshore, wave‐exposed sites. Gaps in the heat map illustrate differences. For example, Madagascan sites were most dissimilar from other countries through a cluster of species that were more abundant in Madagascar and in very low densities or absent elsewhere: the surgeonfish *Acanthurus xanthopterus, A. blochii, A. dussumerii, *the coral grouper *Plectropomus punctatus, *the butterflyfish *Chaetodon vagabundus,* *C. lineolatus*, and the parrotfish *Hipposcarus harid *(first column, Figure 2). Both Madagascar and Comoros also differed due to several species that occurred in very low numbers or were missing, most notably the caesionids (*Caesio* spp.) which were found in most sites throughout Tanzania and Mozambique, and were highly abundant in some of these sites (Figure 2). Other species that occurred in low densities in Madagascar and Comoros were the pennant butterflyfish *Heniochus acuminatus* and the emperor *Lethrinus erythracanthus*. Note that for ease, sites in a country are referred to simply with the country name, but this does not infer that these sites are representative of the country as a whole.

**Figure 3:**
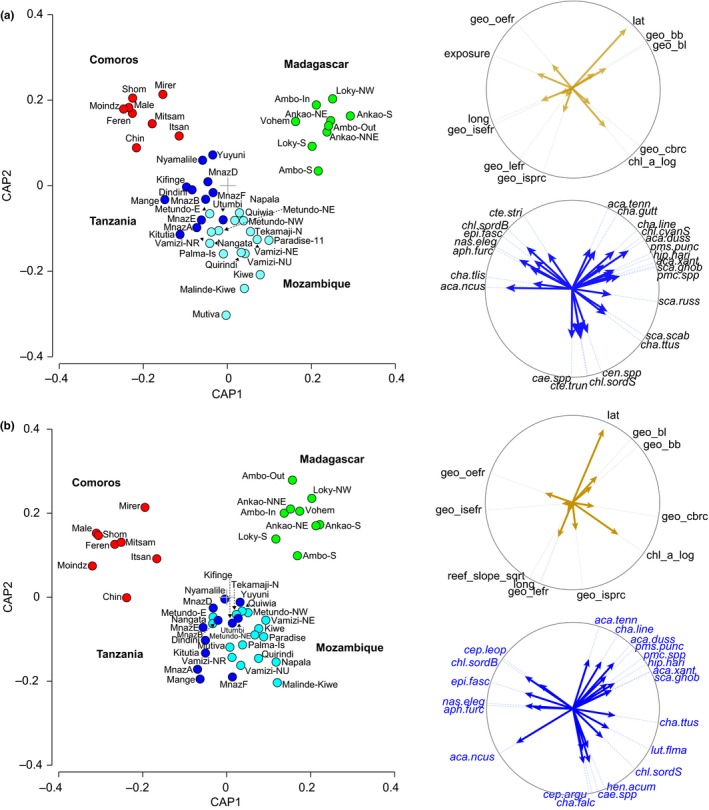
CAP ordination of (a) fish density and (b) fish biomass with (i) vectors indicating the relationship between significant* spatial and environmental variables and the fish assemblages from all 4 countries; and (ii) vectors indicating the fish species most influential in delineating differences between fish assemblages from all four countries (Pearson correlation >0.45). * Significance tested via IndSpecies package in R

**Figure 4:**
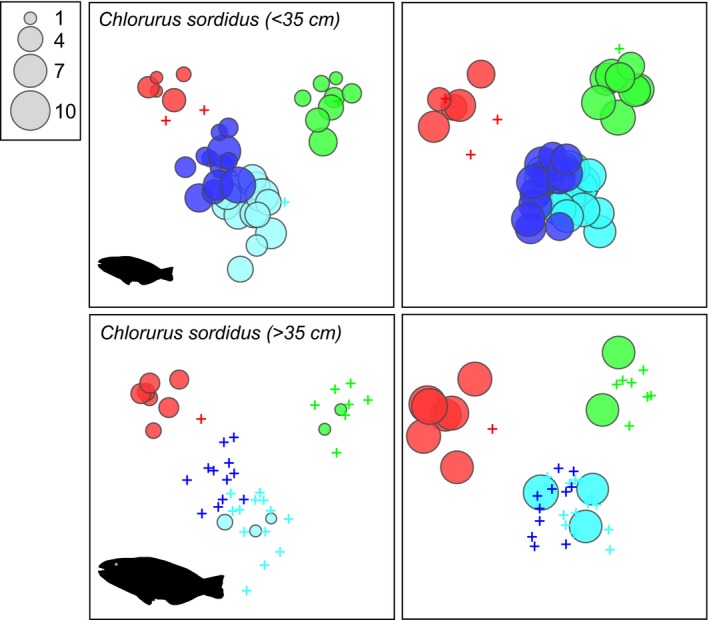
CAP ordinations of density (left) and biomass (right) of fish assemblages with bubble plots representing density indiv./1,000 m^2^ and biomass (kg/1,000 m^2^) of the excavating parrotfish *Chlorurus sordidus* partitioned by size: B (>35 cm) (bottom) and S (<36 cm) (top)

The one‐way PERMANOVA confirmed significant differences between countries in the fish species density data (Pseudo‐*F* = 4.524, *p* = 0.001) and biomass data (Pseudo‐*F* = 4.728, *p* = 0.0001) and the CAP analysis identified clear separation of fish assemblages between countries (Figure 3) with few misclassification errors (Supporting Information Appendix S1). Canonical correlations were high with the first axis separating Madagascar from the others and the second axis separating Comoros, Tanzania, and Mozambique from each other (Figure 3). We identified 24 species as highly influential in describing density patterns (Figure 3a, see vectors on CAP) and 21 species for biomass patterns (Figure 3b), based on a decision rule of Pearson correlation coefficients being |>0.45|. For example, *Acanthurus nigrofuscus* was more abundant at sites in Tanzania and Comoros, *Acanthurus dussumierii *was most abundant in Madagascar, and *Caesio* species were most abundant in Mozambique. A shift in the relative biomass and density of the excavating parrotfish *Chlorurus sordidus *was seen with small individuals (S, <36 cmTL) being more abundant in Mozambique, whereas large *C. sordidus* (B, >35 cmTL) were more abundant in Comoros (Figure 4), suggesting a possible refuge from fishing in Comoros. One of the most abundant and ubiquitous species recorded throughout the region was the small brown detritivorous surgeonfish, *Ctenochaetus striatus, *with maximum densities of 145 indiv./1,000 m^2^ (±83 *SD*) in Shomoni, Grande Comore. This species was highly significant in defining the fish community in all countries based on the SIMPER analysis of density data (Table 4). These mirror patterns in the heat map (Figure 2).

**Table 4:** SIMPER tables of abundance and biomass highlighting the 10 most significant species delineating differences between countries

Country	Comoros	Madagascar	Mozambique	Tanzania
***Abundance***				
Comoros		**65.76**	**59.96**	**59.21**
Madagascar	*Acanthurus nigrofuscus (6.1)*		**62.25**	**62.89**
	Pomacanthid spp*. (1.8)*			
	*Naso elegans (2.6)*			
	*Chlorurus sordidus B (1.7)*			
	*Centropyge *spp*. (1.6)*			
	*Chaetodon meyeri (1.3)*			
	*Chlorurus sordidus S (2.0)*			
	*Acanthurus dussumieri (1.6)*			
	*Acanthurus tennenti (1.7)*			
	*Ctenochaetus striatus (3.1)*			
Mozambique	*Chlorurus sordidus B (1.7)*	*Acanthurus dussumieri (1.5)*		**56.28**
	*Naso elegans (2.4)*	*Acanthurus nigrofuscus (3.4)*		
	*Centropyge *spp*. (2.3)*	*Ctenochaetus striatus (2.6)*		
	*Chlorurus sordidus S (4.1)*	Pomacanthid spp. (1.3)		
	*Chaetodon meyeri (1.2)*	Balistid benthic (1.2)		
	*Ctenochaetus striatus (3.9)*	*Chlorurus sordidus S (3.0)*		
	*Acanthurus nigrofuscus (3.4)*	*Acanthurus tennenti (1.6)*		
	Pomacanthid spp*. (1.2)*	*Centropyge *spp*. (1.8)*		
	*Aphareus furca (1.5)*	*Lutjanus fulviflamma (1.6)*		
	*Ctenochaetus truncatus (2.6)*	*Hipposcarus harid (1.1)*		
Tanzania	*Chlorurus sordidus B (1.9)*	*Acanthurus nigrofuscus (3.0)*	Balistid benthic (2.2)	
	Balistid benthic *(2.4)*	*Cephalopholis argus (1.2)*	*Centropyge *spp*. (1.6)*	
	*Cephalopholis argus (1.2)*	Balistid benthic (1.8)	*Chlorurus sordidus S (2.9)*	
	*Acanthurus nigrofuscus (3.3)*	Pomacanthid spp. (1.6)	*Ctenochaetus striatus (2.6)*	
	*Centropyge *spp*. (1.4)*	*Centropyge *spp*. (1.5)*	Pomacanthid spp*. (1.3)*	
	*Ctenochaetus striatus (3.5)*	*Acanthurus dussumieri (1.4)*	*Cephalopholis argus (0.9)*	
	*Naso elegans (2.0)*	*Acanthurus tennenti (1.6)*	*Acanthurus nigrofuscus (2.0)*	
	*Chaetodon meyeri (1.1)*	*Hipposcarus harid (1.1)*	*Lutjanus fulviflamma (3.0)*	
	*Chlorurus sordidus S (3.4)*	*Lutjanus fulviflamma (3.0)*	*Ctenochaetus truncatus (2.5)*	
	*Aphareus furca (1.5)*	*Chlorurus sordidus S (2.5)*	*Naso elegans (1.2)*	
***Biomass***				
Comoros		**64.2**	**57.3**	**57.2**
Madagascar	*Acanthurus nigrofuscus (2.6)*		**59.2**	**60.3**
	*Naso elegans (2.3)*			
	*Acanthurus dussumieri (2.3)*			
	*Hipposcarus harid (2.2)*			
	*Scarus *spp*. (1.9)*			
	*Chlorurus sordidus B (2.1)*			
	*Pomacanthid *spp*. (1.6)*			
	*Chaetodon meyeri (1.1)*			
	*Scarus tricolor (1.8)*			
	*Aphareus furca (1.6)*			
Mozambique	*Chlorurus sordidus B (2.5)*	*Acanthurus nigrofuscus (2.0)*		**53.5**
	*Ctenochaetus truncatus (1.9)*	*Acanthurus dussumieri (2.3)*		
	*Naso elegans (2.1)*	*Ctenochaetus truncatus (1.7)*		
	*Cephalopholis argus (1.9)*	*Acanthurus tennenti (2.0)*		
	*Scarus *spp*. (1.8)*	*Hipposcarus harid (1.8)*		
	*Chaetodon meyeri (1.0)*	*Cephalopholis argus (1.7)*		
	*Caesio *spp*. (2.3)*	*Scarus tricolor (1.6)*		
	*Aphareus furca (1.8)*	*Chaetodon lineolatus (1.0)*		
	Pomacanthid spp. *(1.4)*	*Scarus *spp*. (1.1)*		
	*Plectorhinchus gaterinus (1.8)*	*Caesio *spp*. (2.0)*		
Tanzania	*Chlorurus sordidusB (2.7)*	*Cephalopholis argus (2.0)*	*Ctenochaetus truncatus (1.7)*	
	*Cephalopholis argus (2.3)*	*Acanthurus nigrofuscus (2.0)*	*Naso elegans (1.7)*	
	*Aphareus furca (1.7)*	*Acanthurus tennenti (2.1)*	*Scarus *spp*. (1.6)*	
	*Chaetodon meyeri (0.9)*	*Acanthurus dussumieri (2.0)*	Pomacanthid spp*. (1.5)*	
	*Acanthurus blochii (1.5)*	*Scarus *spp*. (1.4)*	*Chaetodon meyeri (0.7)*	
	Pomacanthid spp*. (1.4)*	*Scarus tricolor (1.5)*	*Caesio *spp*. (1.9)*	
	*Acanthurus tennenti (1.6)*	*Naso elegans (1.6)*	*Plectorhinchus gaterinus (1.7)*	
	*Scarus *spp*. (1.6)*	Pomacanthid spp*. (1.6)*	*Acanthurus blochii (1.5)*	
	*Naso elegans (1.5)*	*Hipposcarus harid (1.6)*	*Hipposcarus harid (1.4)*	
	*Hipposcarus harid (1.4)*	*Acanthurus blochii (1.4)*	*Heniochus acuminatus (1.3)*	

Notes

Species rankings were ordered by the ratio of species dissimilarity/standard deviation to highlight those species which best highlight the differences between countries. Numbers in parentheses are the percentage contribution of each species to the overall dissimilarity, which is presented in bold type. B: big; S: small.

**Figure 5:**
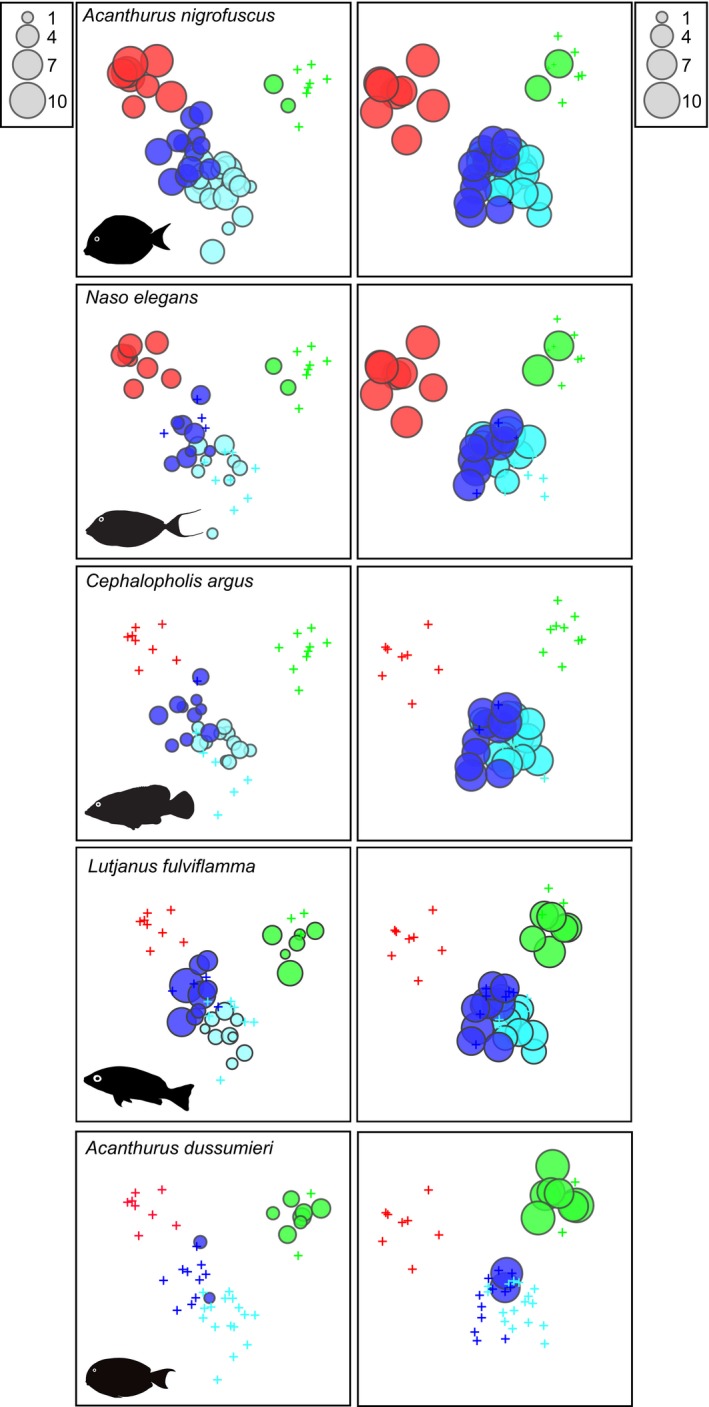
Bubble plots of those species identified as significant delineators of country differences across both abundance (left plots) and biomass (right plots) CAP ordinations. Size of bubbles are comparable within each column and represent square‐root abundances (individ./1,000 m^2^) and log(*x* + 1) biomass (kg/1,000 m^2^), respectively. + = zero count

Significant species that explained regional patterns in fish biomass (Figure 3b) included many of the same species in the analyses for fish density (Table 4). However, some new species appear which were larger bodied, such as the omnivore *Lutjanus fulviflamma* and the piscivorous grouper *Cephalopholis argus*, while others such as *Ctenochaetus striatus* were no longer significant (Figure 3b). The SIMPER analysis with biomass data revealed other significant species, such as the omnivore *Plectorhinchus gaterinus, *the scraper *Scarus tricolor,* the grazer‐detritivore *Acanthurus blochii*, the planktivore *Heniochus acuminatus*, and the corallivore *Chaetodon lineatus* (Table 4). Six species that strongly delineate differences in both density and biomass of species assemblages between the countries are illustrated in Figures 4 and 5.

### 3.2 Abiotic and biotic factors affecting species density and biomass

The most parsimonious DistLM selected four significant variables which explained 39.3% of the observed variance in the fish species density dataset. Space (composite of latitude and longitude) and reef geomorphology were the most significant, explaining 12.8% and 18.9% of the variation in the dataset, respectively. Reef exposure (4.1%) and chlorophyll_*a* (3.5%) were also significant, but at a reduced level. The direction of these variables on the species’ abundance matrix is shown as vectors in the CAP ordination (Figure 3a). Space clearly separates the relative density of species in the assemblages of the two island countries, and from the mainland countries. The vectors show that the 7 reef geomorphology types (Table 1) correlated strongly with fish assemblages at different sites and countries. For example, “Coastal barrier reef complex” (geo_cbrc) reefs were typical of Mozambique, both Mozambique and Tanzania shared fish assemblages characteristic of “Inner seas patch reef complex” (geo‐isprc) reefs, while “Ocean‐exposed fringing reef” (geo_oefr) correlated most with sites in Comoros. The most exposed sites (exposure) were found in Comoros and sites with the highest chlorophyll_*a* were in Mozambique (Figure 3).

The best‐fit DistLM identified similar clear separation of fish assemblages between countries based on biomass data (Figure 3b), explaining 40.4% of the variation in the data and identified three of the same variables as in the density data. Space and geomorphology explained 13.2% and 18.9% of the variation in the dataset, respectively, and Chlorophyll_*a* explained 5.0% of the variation. However, reef slope rather than exposure was significant, explaining 3.3% of the variation in the data. In summary, the CAP analyses and DistLM showed that space (latitude/longitude), reef geomorphology, chlorophyll_*a*, exposure, and slope were valid predictors of the structure of the fish assemblages across the region. Notably, none of the benthic variables, including live coral cover, were significant.

### 3.3 Tanzania and Mozambique datasets

At the smaller spatial scale of Tanzania and Mozambique, the DistLM found geomorphology explained the highest variation in the density data at 23.3%, with exposure and fishing pressure also significant explanatory variables, at 6.5% and 6.0%, respectively. The same variables were also significant correlates of the biomass data with geomorphology, exposure, and fishing pressure explaining 22.7%, 6.5%, and 6.5% of the variance, respectively. Thus, at this smaller spatial scale, fishing pressure became a significant variable for both fish density and biomass. However, again, none of the benthic variables were significant.

### 3.4 Total fish biomass

**Figure 6:**
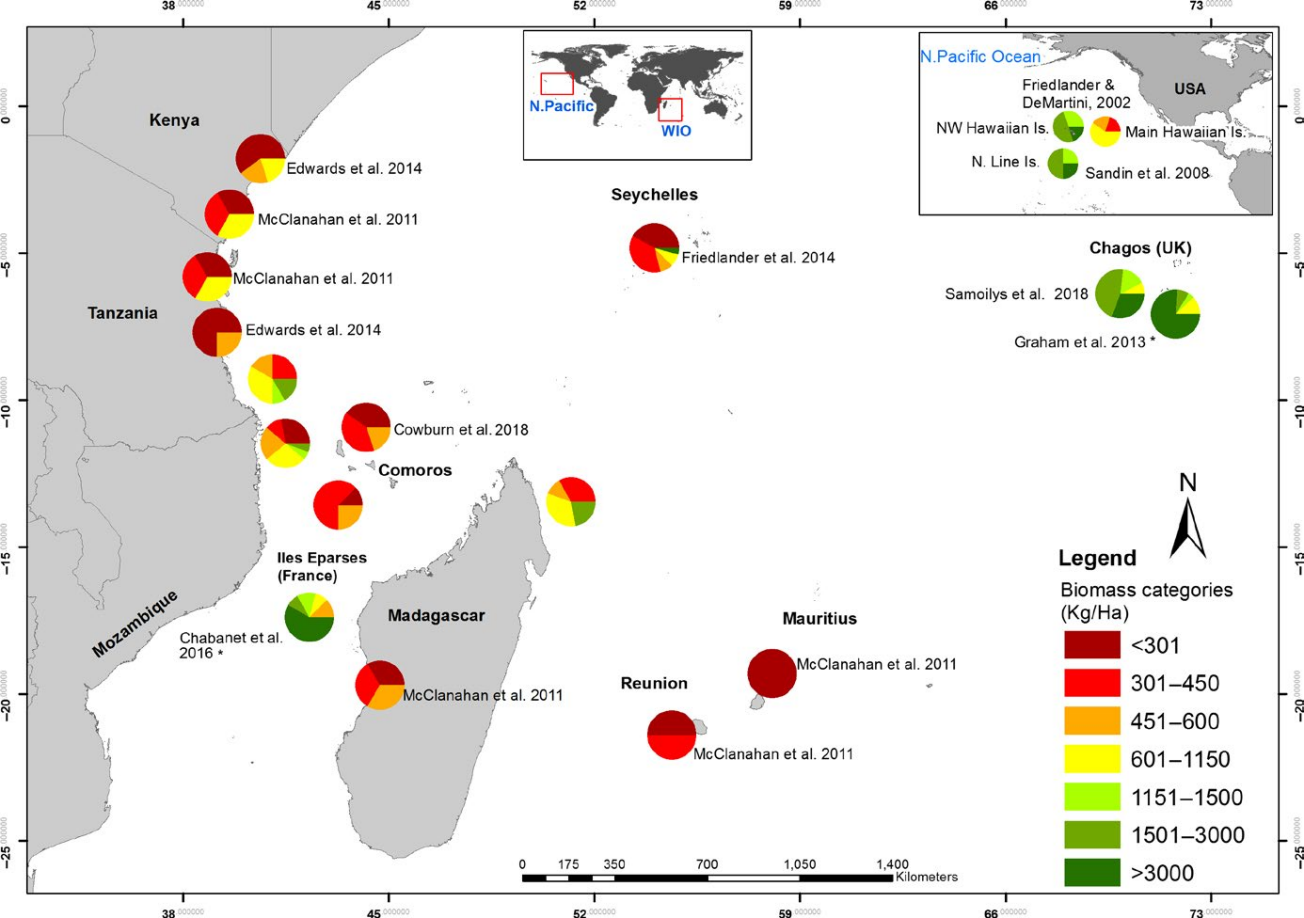
Pie charts representing total biomass (kg/ha) allocated into seven biomass categories based on number of sites per country. Unmarked charts are from 13 families of reef‐associated fishes from this study; other charts represent other studies from the same countries in approximately the same time period. Chagos and the Hawaiian Islands included for comparisons of unfished or lightly fished reefs. *Study's values include sharks

Total fish biomass, based on all 12 families, was low to moderate in all sites in the Comoros at <558 kg/ha and highly variable within the other three countries depending on the location of the survey site (Figure 6, Supporting Information Table S2). For example, of the full set of 21 sites in Mozambique, four sites, some of which were fished, had very high mean biomass values at 1,513–2,306 kg/ha and six sites had high biomass levels at 768–986 kg/ha, while five sites had moderate biomass at 453–544 kg/ha (Supporting Information Table S4). A similar wide range of values was also seen in Tanzania (Supporting Information Table S4).

## 4 DISCUSSION

### 4.1 Geographic patterns in fish assemblages

Geographic location and reef geomorphology were the most significant drivers of the observed patterns in the structure of reef fish assemblages across the WIO, based on density and biomass data across four countries. The largest differences were between sites in the island countries, Comoros and Madagascar, and sites in the mainland eastern Africa countries of Tanzania and Mozambique, suggesting that historic large‐scale geological processes (e.g., Audru et al., 2010; Obura, 2015) are major drivers in structuring coral reef fish assemblages in this region. Other biogeographic processes may also explain the patterns, though our survey sites cannot be seen as representative of each country as a whole, particularly Madagascar. Our results align with some studies from Pacific reefs which found biogeography and/or geomorphology were significant variables in structuring fish assemblages (Heenan et al., 2016; Pinca et al., 2012; Taylor et al., 2014), though contrast with D'agata et al. (2014), who found human influences were more significant drivers than biogeographic variables on parrotfish functional and phylogenetic diversities. We show that the structure of fish assemblages of Comoros, NE Madagascar, northern Mozambique, and Tanzania aligns with ocean‐exposed fringing reefs, lagoon and barrier banks, coastal barrier reef complexes, and inner‐seas‐exposed fringing reefs. Three other biophysical variables were also significant, albeit to a lesser degree: chlorophyll_*a*; reef exposure and reef slope. Since the WIO represents a biogeographic province of similarity in fish species distributions (Bellwood & Wainwright, 2002; Kulbicki, Parravacini, & Mouillot, 2015), the results provide evidence that a reef's geographic location, structure, and surrounding environmental conditions are key variables influencing patterns in reef fish assemblages in the WIO.

### 4.2 Large‐scale environmental drivers of reef fishes

Biogeographic drivers, such as the mid‐domain effect (MDE), reef location, isolation, and connectivity (Mora, 2015; Parravicini et al., 2013), may help explain why the fish assemblages in Comoros and Madagascar are distinctly different from mainland eastern Africa. Other key explanatory variables these studies report include sea surface temperature (SST), coast length, and reef area (see also Bellwood, Hughes, Connolly, & Tanner, 2005), based on predictors of reef fish species richness. While these studies conclude that productivity (chlorophyll_*a*) is not a key factor (Mora, 2015), our results show that chlorophyll_*a* is a significant variable in driving the observed patterns in fish assemblages. Higher productivity on the mainland African coast may support higher densities and biomass of reef fishes. This finding is consistent with a large‐scale study across the Pacific which found higher oceanic productivity was associated with over double the biomass of all reef fishes (sharks and trevally not included), and was notably significant for planktivores and piscivores (Williams et al., 2015). Average composite values (2010–2015) of SST varied by ~1°C between Comoros and the other three countries (27.88°C, 27.89°C, 28.02°C, 26.91°C, for Mozambique, Tanzania, Comoros, Madagascar, respectively, NASA, 2014); therefore, SST may also be a contributing factor to the regional patterns detected. Coast length and reef area, both associated with higher species richness and fish size (Bellwood et al., 2005; Kulbicki et al., 2015; Parravicini et al., 2013), may explain differences between Comoros and the other three countries, since Comoros’ reef area ranges from only 6% to 9% of that of the other three countries, and Comoros’ coast length is between 9% and 34% of the other countries (UNEP‐WCMC, 2010; Wessel & Smith, 1996). For both these variables, Madagascar has the largest reef area and longest coast length; therefore, MDE, isolation, and connectivity may be more important in explaining the strong separation of Madagascan fish assemblages from Tanzania and Mozambique.

### 4.3 Effects of fishing on fish assemblages

Fishing has been shown to precipitate top‐down and bottom‐up trophic cascades on coral reefs (DeMartini et al., 2008; Graham et al., 2017; Sandin & Zgliczynski, 2015). We did not find a significant fishing effect on the fish assemblages we surveyed at the largest spatial scale, despite including two uncorrelated measures of fishing effects: human population density (Cinner, et al., 2009; Taylor et al., 2014) and an index of fishing pressure (Burke et al., 2011). Nor did we detect a MPA protection effect. However, when tested at a smaller spatial scale (Tanzania and Mozambique only), we found fishing pressure explained 6% of the variation in fish assemblages. Nevertheless, at this smaller spatial scale, reef geomorphology remained the overwhelming driver of differences between sites. Two factors may help explain the apparent lack of fishing effects. Firstly, by controlling for spatial correlation, we extracted the most unconfounded fishing signal possible. Secondly, we integrated our surveys over a much greater depth range (0.5–33 m) than other studies (often <15 m) which increased the available biomass in our calculations. If reef productivity is to be measured in fish biomass/per area, we believe it is logical to include the full extent of the reef. In addition, these depths are accessible to fishing by coastal communities. Further, the ~30 m limit imposed by SCUBA diving already restricts accurate measures of target fishery species’ biomass (Lindfield, Harvey, Halford, & McIlwain, 2016). Our results suggest that the fish biomass on a reef is firstly determined by large‐scale factors of geography, geomorphology, and nutrient availability which therefore need to be considered when examining fishing effects or ecosystem functioning (DeMartini & Smith, 2015; Mora et al., 2011).

### 4.4 Fish biomass as an indicator of reef productivity

Total fish biomass is regularly used as an index of productivity on coral reefs and is a more sensitive indicator of fishing effects than density (Graham et al., 2017; McClanahan et al., 2016). However, not all reefs are equal in their ability to sustain high levels of biomass (Williams et al., 2015). Williams and co‐authors attribute large differences in fish biomass in remote unfished reefs in the US Line Islands and NW Hawaii to variable oceanic productivity among locations. In the WIO, we found ocean‐exposed fringing reefs had total fish biomass values of 900–1,100 kg/ha, while lagoon‐exposed fringing reefs and inner seas patch reefs yield moderate total biomass at ~500 kg/ha (Table 5). It is possible that these latter more weather‐protected reefs, and hence more accessible to artisanal fishers, may have reduced biomass due to higher fishing pressure. Smaller spatial scale comparisons are needed to separate these effects. Nevertheless, very high biomass of >1,500 kg/ha was recorded at individual sites in Tanzania, Mozambique, and Madagascar (Figure 6), including sites where there is fishing. These latter values are on a par with some sites in the Chagos Archipelago which is uninhabited and represents close to “pristine” biomass for the WIO (Graham, Pratchett, Mcclanahan, & Wilson, 2013; Samoilys et al., 2018). Our results suggest that the productivity of reefs in the WIO in terms of fish biomass depends on their geomorphology, exposure, and nutrient levels.

**Table 5:** Mean fish biomass (kg/ha ± *SE*) per reef geomorphology (sensu Andréfouët et al., 2009) per country

Geomorphology	bb	bl	cbrc	isefr	isprc	lefr	oefr
*Comoros*				(6)			(24)
Total				448 ± 17			381 ± 8
Pisci/omni				11 ± 4			108 ± 10
Ratio				0.03			0.28
*Madagascar*	(3)	(10)	(10)				(18)
Total	508 ± 17	1,864 ± 51	442 ± 15				995 ± 19
Pisci/omni	36 ± 6	258 ± 27	77 ± 11				366 ± 35
Ratio	0.07	0.14	0.17				0.37
*Mozambique*			(32)	(8)	(17)	(5)	(46)
Total			686 ± 19	834 ± 26	463 ± 12	524 ± 21	1,105 ± 18
Pisci/omni			313 ± 49	180 ± 18	66 ± 8	184 ± 20	374 ± 23
Ratio			0.46	0.22	0.14	0.35	0.34
*Tanzania*				(5)	(15)	(15)	(35)
Total				371 ± 14	647 ± 15	556 ± 10	1,124 ± 23
Pisci/omni				172 ± 7	293 ± 19	108 ± 17	608 ± 37
Ratio				0.46	0.45	0.19	0.54
Mean total by reef type	508 ± 17	1,864 ± 51	628 ± 15	590 ± 13	549 ± 9	548 ± 9	953 ± 10

Note

The surveys sites were restricted to the NE in Madagascar, to N. Cabo Delgado in Mozambique, and to two islands of the Comoros. Mean values presented as total (11 families), piscivores/omnivores (pisci/omni), and ratio of pisci/omni: total. Reef types: bb = bank barrier, bl = bank lagoon, cbrc = coastal barrier reef complex, isefr = inner seas‐exposed fringing reef, isprc = inner seas patch reef, lefr = lagoon‐exposed fringing reef, oefr = ocean‐exposed fringing reef. Numbers in parentheses are total number of transects (replicates) per geomorphology per country. See Supporting Information Table S2 for mean values per site.

Comparing total reef fish biomass between studies can be problematic (Chabanet et al., 2016). For example, studies that include sharks will substantially inflate biomass values up, as illustrated by measures from two studies in Chagos (Figure 6). We suggest the biomass of Epinephelinae, Lutjanidae, Lethrinidae, and Haemulidae (piscivores and omnivores) may be a useful metric, as these families contain widely exploited target fishery species in coral reef fisheries (Samoilys & Carlos, 2000) and have been regularly surveyed in UVC surveys in the WIO over the last 20 years (Obura, et al., 2017). We recorded the highest biomass of piscivores/omnivores on ocean‐exposed fringing reefs in Tanzania, some of which were protected from fishing within the Mafia Island National Marine Park, while similar reef types that were fished in Mozambique yielded half this value (Table 5). We propose that a ratio of piscivores/omnivores to total biomass may provide a useful metric of fishery exploitation and that values ~0.3 represent naturally productive reefs that are fished, and that higher values of ~0.4–0.5 might be achieved through protection within MPAs (Table 5). The latter compare with 0.44–0.52 recorded on atoll rim and lagoon sites, respectively, in the unfished Chagos Archipelago (Samoilys et al., 2018).

### 4.5 Patterns in species assemblages

A relatively small number of species were significant in explaining the differences in fish assemblages between countries. From a sampled assemblage of 123 reef‐associated fishes, just over 30% were significant, including ten species/taxa ubiquitous across the region, though with varying densities: *Chaetodon melannotus* and *C. guttatissimus *(invertivores), *Pomancanthus* spp. (invertivores) and *Centropyge* spp. (grazer‐detritivores), *Sufflamen* spp. (invertivores), and *Acanthurus leucosternon *and *A. nigrofuscus *(grazers), *A. tennenti* (grazer‐detritivores), *Naso elegans *(browser), and *Ctenochaetus striatus *(detritivore). The functional role of these species within the reef ecosystem is likely driven by trophic pathways (Bellwood, Goatley, Brandl, & Bellwood, 2014; Wilson, Bellwood, Choat, & Furnas, 2003). The predominance of lower trophic level species among these taxa suggests bottom‐up influences of detritus, algae, and small invertebrates are important.

Other species significant in delineating differences in assemblage structure are highlighted here because they may serve as useful indicators for reef type and health. These included the detritivorous *Ctenochaetus striatus,* which was most the abundant species throughout the region and is known to prefer low sediment levels on reefs (Tebbett, Goatley, & Bellwood, 2017a, 2017b). We recorded exceptionally high densities at some sites such as Shomoni, Grande Comore (1,452 ± 832 *SD* indiv./ha), and Tekamaji, Mozambique (1,224 ± 872 *SD* indiv./ha), suggesting these sites may represent reefs with crystalline waters. Some species were only seen on the east African mainland such as the small piscivorous grouper, *Cephalopholis argus,* and the planktivorous Caesionidae which are an important food source for piscivores (Hobson, 1991). High densities of caesionids may be related to the higher chlorophyll_*a* levels on the mainland. The snapper*, Lutjanus fulviflamma *was widely distributed and highly abundant at some sites Tanzania and Mozambique but not observed in Comoros. It is likely that few or no mangroves near most sites in Comoros may be the reason for the lack of *L. fulviflamma* since their juvenile phase is almost entirely in mangroves (Kimirei, Nagelkerken, Mgaya, & Huijbers, 2013). The absence of caesionids and *C. argus* at sites in Comoros and Madagascar is not easily explained since both taxa are abundant in the Chagos Archipelago (Samoilys et al., 2018; Winterbottom & Anderson, 1997), but possibly island biogeography, reef area, and connectivity may play a role (Bellwood et al., 2005; Mora, 2015; Parravicini et al., 2013; Sandin, Vermeij, & Hurlbert, 2008). A small group of species were observed in NE Madagascar that were either rare or absent elsewhere which included three grazing‐detritivore acanthurids (sensu Green & Bellwood, 2009): *A. dussumieri, A. blochii*, and *A. xanthopterus* and the grouper *Plectropomus punctatus*. Again, the influence of detritus and algae is suggested by these acanthurids. *Plectropomus punctatus* is a widespread grouper endemic to the Indian Ocean that is susceptible to fishing pressure, suggesting this may be the reason that it was uncommon or unsighted in Comoros, Tanzania, and Mozambique. The distribution pattern of the size and hence trophic group of the widely distributed excavator, *Chlorurus sordidus,* may also reflect fishing pressure. Larger individuals were more abundant in Comoros and smaller individuals more abundant in Mozambique, possibly because parrotfishes are not targeted in Comorian artisanal fisheries (Freed & Granek, 2014). The potential for the species discussed here to serve as candidate biodiversity indicator species for monitoring coral reef health, for example, by the Convention on Biological Diversity (Pereira et al., 2013) is an important avenue for further research.

Climate change‐induced coral bleaching is unquestionably one of the primary impacts occurring on coral reefs today (Hughes et al., 2018) with concomitant impacts on reef fish assemblages due to the loss of live coral (Graham et al., 2007). There is a substantial body of work that demonstrates clear relationships between reef benthos and the structure of reef fish assemblages with the food and shelter provided by coral invoked to explain these relationships. The most widely understood relationship is between obligate corallivore butterflyfish species and the extent of live coral (Munday, Jones, Pratchett, & Williams, 2008) and two corallivorous chaetodons, *Chaetodon meyeri *and *C. lineolatus,* were among the significant species in delineating the differences in assemblages between countries. It is therefore surprising that none of the six benthic variables in our study were significant in explaining patterns in fish assemblages, even at the smaller scale of mainland Africa. In contrast, significant associations between live coral cover, recently dead coral, and rugosity and the structure of fish assemblages were found in the Chagos Archipelago (Samoilys et al., 2018). The different outputs from these studies support the view that the influence of benthic variables on fish communities is scale dependent and that confounding variables such as geography, geomorphology, and fishing pressure need to be controlled.

## 5 CONCLUSIONS

The observed differences in the structure of fish assemblages across the WIO support the growing understanding that comparing reef fish assemblages across large spatial scales has to first take into account reef geomorphology, other reef structural attributes, and larger scale environmental drivers such as nutrient levels (Heenan et al., 2016; Mora, 2015; Taylor et al., 2014; Williams et al., 2015). Understanding the effects of fishing or loss of live coral from bleaching on fish assemblages needs to be examined where these larger scale variables are explicitly controlled for, which is often at smaller scales. We show a subset of species responded strongly to environmental conditions, though a large number did not. The role these significant species may play as biodiversity indicators for coral reefs, and the trophic dynamics of these assemblages are important avenues for future research. We suggest that variation in fish assemblages caused by the extent of reef area and coastline (Bellwood et al., 2005; Parravicini et al., 2013) is still poorly understood, and the eastern African coastline provides an ideal location for further research. However, our models revealed 60% of the variation in fish assemblages remains unexplained. This highlights an urgent need to develop more spatially structured and controlled monitoring programmes with better measures of fishing pressure if we are to properly understand the influence of anthropogenic effects on coral reef systems.

## CONFLICT OF INTEREST

None declared.

## AUTHOR CONTRIBUTIONS

MAS conceived the ideas. MAS collected the data. AH led the data analysis. MAS led the writing. AH contributed to the writing. KO contributed ideas, data analysis, and writing.

## Data Availability

Data analyzed in this paper are archived in open format on Dryad (https://doi.org/10.5061/dryad.st27t1k).
