# Corrigendum

**DOI:** 10.1002/ece3.5546

**Published:** 2019-08-22

In “Disentangling drivers of the abundance of coral reef fishes in the Western Indian Ocean,” which was published in volume 9 issue 7, April 2019, the word Comoros should be changed to Madagascar in the Discussion section 4.2. The correct information is printed below.

Average composite values (2010–2015) of SST varied by ~1°C between Madagascar and the other three countries (27.88°C, 27.89°C, 28.02°C, 26.91°C, for Mozambique, Tanzania, Comoros, Madagascar, respectively, NASA, 2014).
