# Supplementary material for: Disentangling drivers of the abundance of coral reef fishes in the Western Indian Ocean
Source: Ecol Evol. 2019 Mar 21;9(7):4149–67. doi: 10.1002/ece3.5044 (PMC6468081; doi:10.1002/ece3.5044)
Supplement: Supplementary file 1 [file ECE3-9-4149-s001.docx]

Samoilys, et al. WIO reef fish assemblages

Supplementary Figure S1.

**
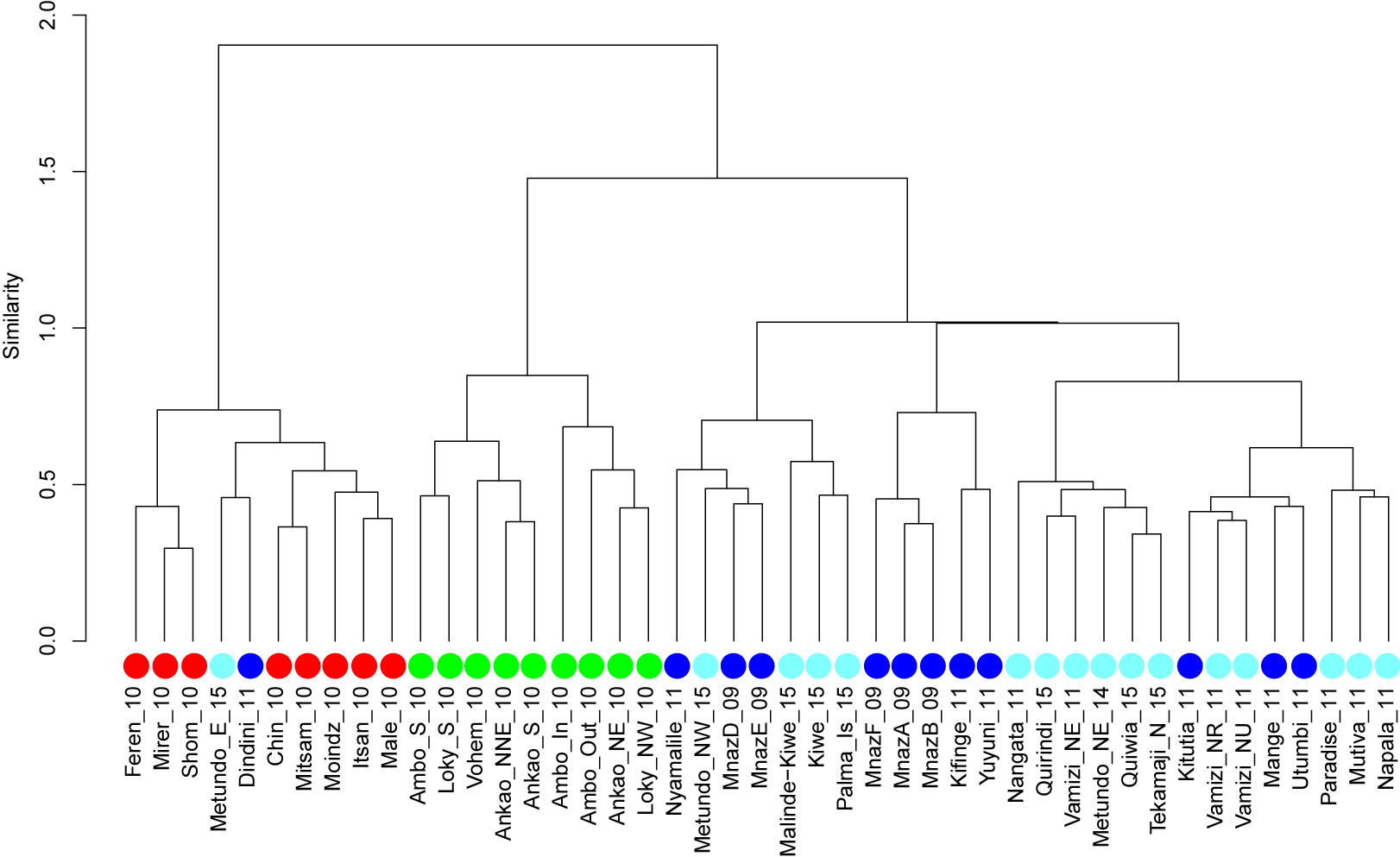
**

Figure S1. Bray-Curtis cluster analysis on 123 species showing 5 groupings with Comoros (red) and Madagascar (green) sites separating clearly. Sites in Tanzania (dark blue) and Mozambique (turquoise) are more similar, spread across the other 3 groupings.
