# Supplementary material for: Disentangling drivers of the abundance of coral reef fishes in the Western Indian Ocean
Source: Ecol Evol. 2019 Mar 21;9(7):4149–67. doi: 10.1002/ece3.5044 (PMC6468081; doi:10.1002/ece3.5044)
Supplement: Supplementary file 3 [file ECE3-9-4149-s003.docx]

**Table S2.** Categories used to define survey sites based on exposure to oceanic conditions, depth, rugosity, benthos and reef profile. CCA=crustose calcareous algae.

Degree of reef slope estimated visually.

| *Exposure to open ocean/waves* | | *Depth category* | |
| --- | --- | --- | --- |
| bay | 1 | shallow | 0.5-7.0m |
| semi-protected inner reef complex or N/NW facing with some protection eg. from island, land mass | 2 | mid-depth | 8.0-15.0m |
| open sea facing N/NW/W | 3 | deep | 9- >15 m |
| open sea facing S/SE/E with some protection from trade winds (eg submerged reef or bank or land mass further offshore) | 4 | high range mid depth | 0.5-15.0m |
| facing S/SE/E with full exposure to trade winds & oceanic conditions | 5 | high range deep | 2.0-33m |
| *Rugosity & reef slope* | | ***Benthos*** | |
| low | 1 | Hard coral | % cover |
| medium | 2 | Fleshy macro-algae | % cover |
| moderately high | 3 | Turf algae | % cover |
| very high | 4 | CCA | % cover |
| Slope | Degree | rubble | % cover |
