# Supplementary material for: Disentangling drivers of the abundance of coral reef fishes in the Western Indian Ocean
Source: Ecol Evol. 2019 Mar 21;9(7):4149–67. doi: 10.1002/ece3.5044 (PMC6468081; doi:10.1002/ece3.5044)
Supplement: Supplementary file 4 [file ECE3-9-4149-s004.docx]

**Table S3.** Level of protection index developed for study sites. MPA=marine protected area; LMMA = locally managed marine area; NTZ= no take zone.

| Protection index | Description |
| --- | --- |
| 1. None | No management in place at all |
| 2. Low | Site considered for protection – eg. MPA proposed. Indicates interest and concern but on the ground management not in place, or MPA in place but no enforcement |
| 3. Moderate | MPA gazetted and established; effectiveness weak – poor enforcement, eg. community reserve with some protection |
| 4. Medium | Some restriction and enforcement, informal legislation. e.g. Tourism zone |
| 5. High | MPA or LMMA in place, enforcement good, some NTZs – if whole site is NTZ and enforced then classified as 6 |
| 6. Very high | Park or NTZ, strongly enforced |
