# Supplementary material for: Disentangling drivers of the abundance of coral reef fishes in the Western Indian Ocean
Source: Ecol Evol. 2019 Mar 21;9(7):4149–67. doi: 10.1002/ece3.5044 (PMC6468081; doi:10.1002/ece3.5044)
Supplement: Supplementary file 5 [file ECE3-9-4149-s005.docx]

**Table S4.** Mean fish biomass (kg/ha±SE) and number of transects (n) per reef geomorphology per site and country. Mean values presented as total (11 families). Reef types: bb= bank barrier, bl=bank lagoon, cbrc=coastal barrier reef complex, isefr = Inner-seas exposed fringing reef, isprc=inner seas patch reef, lefr=lagoon exposed fringing reef, oefr= ocean-exposed fringing reef.

| **Reef geomorphology** | **bb** | |  | **bl** | | | **cbrc** | | | **isefr** | | | **isprc** | | | **lefr** | | | **oefr** | | |
| --- | --- | --- | --- | --- | --- | --- | --- | --- | --- | --- | --- | --- | --- | --- | --- | --- | --- | --- | --- | --- | --- |
| **Country/site** | **Mean** | **SE** | **n** | **Mean** | **SE** | **n** | **Mean** | **SE** | **n** | **Mean** | **SE** | **n** | **Mean** | **SE** | **n** | **Mean** | **SE** | **n** | **Mean** | **SE** | **n** |
| **Comoros** |  |  |  |  |  |  |  |  |  | **448.4** | **17.1** | **6** |  |  |  |  |  |  | **381.1** | **7.7** | **24** |
| Chin |  |  |  |  |  |  |  |  |  |  |  |  |  |  |  |  |  |  | 516.6 | 22.2 | 5 |
| Feren |  |  |  |  |  |  |  |  |  | 558.1 | 32.4 | 3 |  |  |  |  |  |  |  |  |  |
| Itsan |  |  |  |  |  |  |  |  |  |  |  |  |  |  |  |  |  |  | 286.2 | 24.3 | 5 |
| Male |  |  |  |  |  |  |  |  |  |  |  |  |  |  |  |  |  |  | 400.5 | 16.69 | 3 |
| Mirer |  |  |  |  |  |  |  |  |  | 338.6 | 11.0 | 3 |  |  |  |  |  |  |  |  |  |
| Mitsam |  |  |  |  |  |  |  |  |  |  |  |  |  |  |  |  |  |  | 312.6 | 8.6 | 5 |
| Moindz |  |  |  |  |  |  |  |  |  |  |  |  |  |  |  |  |  |  | 448.4 | 14.2 | 3 |
| Shom |  |  |  |  |  |  |  |  |  |  |  |  |  |  |  |  |  |  | 340.7 | 15.7 | 3 |
| **Madagascar** | **508.4** | **16.9** | **3** | **1864.0** | **51.0** | **10** | **442.5** | **14.5** | **10** |  |  |  |  |  |  |  |  |  | **995.9** | **19.0** | **18** |
| Ambo Inner |  |  |  |  |  |  |  |  |  |  |  |  |  |  |  |  |  |  | 658.1 | 42.3 | 3 |
| Ambo Outer |  |  |  |  |  |  |  |  |  |  |  |  |  |  |  |  |  |  | 329.8 | 26.0 | 5 |
| Ambo S |  |  |  |  |  |  |  |  |  |  |  |  |  |  |  |  |  |  | 1046.1 | 22.2 | 5 |
| Ankao NE | 508.4 | 16.9 | 3 |  |  |  |  |  |  |  |  |  |  |  |  |  |  |  |  |  |  |
| AnkaoNNE |  |  |  | 709.7 | 23.9 | 5 |  |  |  |  |  |  |  |  |  |  |  |  |  |  |  |
| AnkaS |  |  |  | 3018.4 | 94.3 | 5 |  |  |  |  |  |  |  |  |  |  |  |  |  |  |  |
| Loky S |  |  |  |  |  |  |  |  |  |  |  |  |  |  |  |  |  |  | 1814.3 | 47.9 | 5 |
| LokyNW |  |  |  |  |  |  | 433.2 | 28.0 | 5 |  |  |  |  |  |  |  |  |  |  |  |  |
| Vohem |  |  |  |  |  |  | 451.8 | 13.2 | 5 |  |  |  |  |  |  |  |  |  |  |  |  |
| **Reef geomorphology** | **bb** | |  | **bl** | | | **cbrc** | | | **isefr** | | | **isprc** | | | **lefr** | | | **oefr** | | |
| **Country/site** | **Mean** | **SE** | **n** | **Mean** | **SE** | **n** | **Mean** | **SE** | **n** | **Mean** | **SE** | **n** | **Mean** | **SE** | **n** | **Mean** | **SE** | **n** | **Mean** | **SE** | **n** |
| **Mozambique** |  |  |  |  |  |  | **686.5** | **19.2** | **32** | **833.5** | **25.9** | **8** | **463.0** | **11.9** | **17** | **524.4** | **20.6** | **5** | **1105.0** | **18.3** | **46** |
| Baixo Pinguim NE | |  |  |  |  |  | 808.6 | 54.3 | 4 |  |  |  |  |  |  |  |  |  |  |  |  |
| FernauVloso |  |  |  |  |  |  |  |  |  |  |  |  |  |  |  | 524.4 | 20.6 | 5 |  |  |  |
| Kiwe |  |  |  |  |  |  |  |  |  |  |  |  | 514.2 | 11.3 | 10 |  |  |  |  |  |  |
| Lalane |  |  |  |  |  |  |  |  |  |  |  |  | 74.1 | 11.0 | 2 |  |  |  |  |  |  |
| MakungN |  |  |  |  |  |  | 2114.1 | 205.9 | 3 |  |  |  |  |  |  |  |  |  |  |  |  |
| Malinde-Kiwe |  |  |  |  |  |  |  |  |  |  |  |  | 516.2 | 34.5 | 5 |  |  |  |  |  |  |
| Metundflat |  |  |  |  |  |  | 453.4 | 24.7 | 5 |  |  |  |  |  |  |  |  |  |  |  |  |
| Metundo NW |  |  |  |  |  |  | 214.7 | 5.53 | 5 |  |  |  |  |  |  |  |  |  |  |  |  |
| MetundoE |  |  |  |  |  |  |  |  |  |  |  |  |  |  |  |  |  |  | 985.9 | 52.8 | 3 |
| MetundoNE |  |  |  |  |  |  |  |  |  |  |  |  |  |  |  |  |  |  | 393.6 | 9.3 | 8 |
| Mutiva |  |  |  |  |  |  |  |  |  | 371.2 | 23.8 | 5 |  |  |  |  |  |  |  |  |  |
| Nangata |  |  |  |  |  |  |  |  |  |  |  |  |  |  |  |  |  |  | 1512.5 | 48.2 | 5 |
| Palma Islands |  |  |  |  |  |  | 315.6 | 11.2 | 5 |  |  |  |  |  |  |  |  |  |  |  |  |
| Paradise |  |  |  |  |  |  |  |  |  | 1604.2 | 48.0 | 3 |  |  |  |  |  |  |  |  |  |
| Quifuki1 |  |  |  |  |  |  |  |  |  |  |  |  |  |  |  |  |  |  | 983.4 | 30.6 | 3 |
| Quirindi |  |  |  |  |  |  |  |  |  |  |  |  |  |  |  |  |  |  | 952.1 | 35.3 | 5 |
| Quiwia |  |  |  |  |  |  |  |  |  |  |  |  |  |  |  |  |  |  | 768.1 | 21.5 | 5 |
| TekamajiN |  |  |  |  |  |  | 950.8 | 29.9 | 5 |  |  |  |  |  |  |  |  |  |  |  |  |
| VamiziNE |  |  |  |  |  |  |  |  |  |  |  |  |  |  |  |  |  |  | 2305.8 | 64.8 | 10 |
| VamiziNR |  |  |  |  |  |  |  |  |  |  |  |  |  |  |  |  |  |  | 364.8 | 9.4 | 7 |
| VamiziNU |  |  |  |  |  |  | 543.8 | 16.2 | 5 |  |  |  |  |  |  |  |  |  |  |  |  |

| **Reef geomorphology** | **bb** | |  | **bl** | | | **cbrc** | | | **isefr** | | | **isprc** | | | **lefr** | | | **oefr** | | |
| --- | --- | --- | --- | --- | --- | --- | --- | --- | --- | --- | --- | --- | --- | --- | --- | --- | --- | --- | --- | --- | --- |
| **Country/site** | **Mean** | **SE** | **n** | **Mean** | **SE** | **n** | **Mean** | **SE** | **n** | **Mean** | **SE** | **n** | **Mean** | **SE** | **n** | **Mean** | **SE** | **n** | **Mean** | **SE** | **n** |
| **Tanzania** |  |  |  |  |  |  |  |  |  | **370.5** | **14.1** | **5** | **646.6** | **14.6** | **15** | **555.6** | **10.0** | **15** | **1124.2** | **23.1** | **35** |
| Chumb |  |  |  |  |  |  |  |  |  | 370.5 | 14.1 | 5 |  |  |  |  |  |  |  |  |  |
| Dindini |  |  |  |  |  |  |  |  |  |  |  |  |  |  |  |  |  |  | 1455.8 | 56.1 | 5 |
| Kifinge |  |  |  |  |  |  |  |  |  |  |  |  |  |  |  |  |  |  | 2152.3 | 116.5 | 5 |
| Kitutia |  |  |  |  |  |  |  |  |  |  |  |  | 743.9 | 31.0 | 5 |  |  |  |  |  |  |
| Mange |  |  |  |  |  |  |  |  |  |  |  |  | 565.1 | 26.9 | 5 |  |  |  |  |  |  |
| MnazA |  |  |  |  |  |  |  |  |  |  |  |  |  |  |  |  |  |  | 563.3 | 55.2 | 5 |
| MnazB |  |  |  |  |  |  |  |  |  |  |  |  |  |  |  |  |  |  | 711.7 | 20.2 | 5 |
| MnazD |  |  |  |  |  |  |  |  |  |  |  |  |  |  |  | 321.3 | 9.6 | 5 |  |  |  |
| MnazE |  |  |  |  |  |  |  |  |  |  |  |  |  |  |  | 316.3 | 12.9 | 5 |  |  |  |
| MnazF |  |  |  |  |  |  |  |  |  |  |  |  |  |  |  |  |  |  | 309.0 | 12.2 | 5 |
| Mnem |  |  |  |  |  |  |  |  |  |  |  |  |  |  |  |  |  |  | 1100.6 | 45.1 | 5 |
| Nyamalile |  |  |  |  |  |  |  |  |  |  |  |  | 630.8 | 16.0 | 5 |  |  |  |  |  |  |
| Utumbi |  |  |  |  |  |  |  |  |  |  |  |  |  |  |  | 1029.2 | 21.3 | 5 |  |  |  |
| Yuyuni |  |  |  |  |  |  |  |  |  |  |  |  |  |  |  |  |  |  | 1576.8 | 48.0 | 5 |
