# Supplementary material for: Disentangling drivers of the abundance of coral reef fishes in the Western Indian Ocean
Source: Ecol Evol. 2019 Mar 21;9(7):4149–67. doi: 10.1002/ece3.5044 (PMC6468081; doi:10.1002/ece3.5044)
Supplement: Supplementary file 6 [file ECE3-9-4149-s006.docx]

**Appendix S1. Supporting information on data compilation and analyses**

*Collinearity tests*

Collinearity tests using VIF (Table 1 below) led to two depth measures being used: depth range category (see separate Table S2) and minimum depth. Further, Reef type was strongly correlated with Geomorphology and therefore only Geomorphology was used in the analyses because it represented a broader scale descriptor of the reef and was more evenly spread across all sites. Geomorphology was categorical and therefore re-coded into 7 binary variables. The spatial coordinates, latitude and longitude, were transformed into cartesian coordinates and combined into a variable called “space”.

Table 1. VIF results.

**#test for Variance Inflation Factors to see if there is high collinearity among explanatory variables**

**# if there is then need to perform env variable reduction**

**#run the model with only the env variables**

> fish_env <- rda(fish.h~coral_cover)

+ macroalgae + turf_algae_sqrt + ccc_4thrt + rubble_sqrt + reef_slope_sqrt + rugosity + exposure

+ max_d_inv_sqrt + min_d_sqrt + d_range + chl_a_log + popn_density_4throot + fp + protection

+ geo_oefr + geo_isefr + geo_bl + geo_bb + geo_cbrc + geo_lefr + geo_isprc, data=env)

**#can see that 2 variables have extremely high VIF values (>20) , hence should remove and re-run VIF call to see the change**

vif.cca(fish_env)

coral_cover macroalgae turf_algae_sqrt ccc_4thrt rubble_sqrt reef_slope_sqrt

2.779638 3.138589 2.866262 1.812109 2.541047 2.489604

rugosity exposure max_d_inv_sqrt min_d_sqrt d_range chl_a_log

3.076825 3.928522 35.297730 13.812147 27.040231 3.868908

popn_density_4throot fp protection geo_oefr geo_isefr geo_bl

5.175164 4.093772 4.255543 16.281116 5.201081 3.136507

geo_bb geo_cbrc geo_lefr geo_isprc

2.667941 8.226812 5.192100 7.117243

**# rerun analysis without the 2 variables with high VIF scores**

> fish_env2 <- rda(fish.h~coral_cover

+ macroalgae + turf_algae_sqrt + ccc_4thrt + rubble_sqrt + reef_slope_sqrt + rugosity + exposure

+ min_d_sqrt + chl_a_log + popn_density_4throot + fp + protection

+ geo_oefr + geo_isefr + geo_bl + geo_bb + geo_cbrc + geo_lefr + geo_isprc, data=env)

> vif.cca(fish_env2)

**# can see that the highest VIF score is now 12.6 so this is acceptable.**

coral_cover macroalgae turf_algae_sqrt ccc_4thrt rubble_sqrt reef_slope_sqrt rugosity exposure

2.736406 3.087437 2.662019 1.628166 2.445604 2.289636 2.879392 3.788536

min_d_sqrt chl_a_log popn_density_4throot fp protection geo_oefr geo_isefr geo_bl

2.629658 3.831048 4.643414 4.026782 3.688080 12.604616 4.562327 3.037826

geo_bb geo_cbrc geo_lefr geo_isprc

2.404207 6.556838 4.818102 7.009422

*Mantel correlograms*

Mantel correlograms identified significant spatial autocorrelation in abundance and biomass data at the largest regional scale (4 countries) therefore the group variable Space was forced into all DistLM models as the first term, ensuring that subsequent tests were conditioned on space. We found no evidence of spatial autocorrelation in the reduced dataset of the African mainland countries, Tanzania and Mozambique, hence no explicit spatial variable was added to the model for this reduced dataset.

*CAP analysis*

A CAP analysis was used because a PCO ordination did not delineate the group differences well. The CAP analysis identified clear separation between countries and misclassification errors were low, limited to only 3 sites out of 45. Those that were misallocated were 3 from Tanzania to Mozambique and 1 from Mozambique to Tanzania.

*Distance Based Linear Modelling*

Initial runs of the DistLM procedure for both the abundance and biomass datasets included all explanatory variables, which were tested for significant independent relationships with the species matrices using marginal tests. Apart from spatial correlations, the Geomorphology variable ‘geo’ explained the most significant amount of variance in the fish assemblages by a factor of 3-12 times of any other variable, hence this variable was also forced into the model after space, when space was significant (regional dataset), or as the first variable in the model when space was not significant (mainland countries dataset).

The BEST procedure was used for building the models. This examines the value of the selection criteria for all possible combinations of predictor variables, providing the best model based on 1-16 variables. AIC_c_ was used because it deals with datasets where the number of samples (sites) is small relative to the number of predictor variables. It has been shown to overfit in univariate models while the BIC selection criteria can be overly harsh, potentially removing important variables (Anderson et al., 2008). Thus, explanatory variables selected by both procedures provided the best selection of significant variables. We also chose models with <5 predictor variables to obtain the most parsimonious models and to avoid overfitting, given there were only 45 replicates (sites). See Table 2 below for model coefficients.

References cited:

Anderson, M., Gorley, R., & Clarke, K. (2008). PERMANOVA+ for PRIMER: Guide to software and statistical methods. Plymouth: PRIMER-E Ltd.

Appendix S1-Table 2. Distance based Linear Model coefficients.

**Fish density**

Coefficients for linear combinations of Xs in the formation of dbRDA coordinates)

| Variable | dbRDA1 | dbRDA2 | dbRDA3 | dbRDA4 | dbRDA5 | dbRDA6 | dbRDA7 | dbRDA8 | dbRDA9 | dbRDA10 |
| --- | --- | --- | --- | --- | --- | --- | --- | --- | --- | --- |
| lat | 0.010603 | -0.031168 | -0.00017264 | 0.0095471 | 0.0031159 | -0.0010435 | 0.0025223 | 0.01035 | 0.00071054 | 0.0047024 |
| long | 0.0018737 | -0.014966 | 0.022768 | 0.0027905 | -0.023123 | -0.017194 | -0.015891 | 0.025694 | 0.0091773 | 0.013034 |
| exposure | -4.378 | -1.7242 | 2.6896 | 2.4954 | 6.3995 | 1.4487 | -0.35913 | 0.4118 | -1.3526 | -0.056161 |
| chl_a_log | 11.612 | 3.7345 | 3.8468 | -6.3859 | 7.9089 | -4.2065 | 1.9953 | 3.1385 | -1.7944 | 4.7125 |
| geo_oefr | -9.2843 | -1.426 | -2.7102 | -8.9222 | -4.5384 | -4.5782 | 7.7991 | 1.7945 | 0.070495 | 2.6668 |
| geo_isefr | -1.0345 | -1.0502 | -11.826 | 4.4966 | 0.015636 | -14.765 | -4.2945 | 13.381 | -2.6721 | 5.1994 |
| geo_bl | 5.8077 | 1.1684 | 22.676 | -6.8273 | -8.6778 | -5.995 | -5.9218 | -4.6136 | -10.086 | -10.918 |
| geo_bb | 6.1439 | -1.9649 | -1.8319 | -7.5819 | -1.3579 | 0.052757 | -17.637 | -20.691 | 14.349 | 6.5461 |
| geo_cbrc | 0.82095 | 0.15386 | -4.6929 | -7.3322 | -0.56392 | 0.55388 | 3.1403 | 7.8537 | 5.9316 | -4.0473 |
| geo_lefr | 1.9614 | 0.61795 | -1.8838 | 5.4009 | 12.222 | 21.272 | 4.9412 | 5.4338 | -10.315 | 5.1085 |
| geo_isprc | -4.4152 | 2.5008 | 0.26788 | 20.766 | 2.9006 | 3.4594 | 11.972 | -3.1586 | 2.7221 | -4.5554 |

**Fish Biomass**

(Coefficients for linear combinations of Xs in the formation of dbRDA coordinates)

| Variable | dbRDA1 | dbRDA2 | dbRDA3 | dbRDA4 | dbRDA5 | dbRDA6 | dbRDA7 | dbRDA8 | dbRDA9 | dbRDA10 |
| --- | --- | --- | --- | --- | --- | --- | --- | --- | --- | --- |
| lat | 0.020559 | -0.024822 | 0.0041265 | -0.0011995 | -0.0061632 | -0.0080053 | -0.0029309 | 0.0028284 | 0.0057935 | 0.0045892 |
| long | -0.0042133 | -0.010393 | 0.03531 | 0.0057384 | -0.014654 | -0.031268 | 0.0044458 | -0.0062962 | 0.0039663 | 0.015753 |
| reef_slope_sqrt | -1.1427 | 0.56222 | 3.3367 | -1.9664 | -1.4525 | -0.034597 | -0.29251 | -0.4528 | 0.59592 | -1.2654 |
| chl_a_log | 10.651 | 9.6686 | 10.983 | -7.1653 | 1.6537 | 1.7373 | -1.6921 | 0.28008 | 1.9968 | 4.8265 |
| geo_oefr | -11.616 | -4.7397 | 3.2553 | -1.767 | 3.8232 | 3.7475 | -4.0701 | 4.1029 | 1.5971 | 4.8745 |
| geo_isefr | 0.13646 | -1.5621 | -3.6321 | -13.122 | -9.356 | -10.879 | -5.144 | -1.9072 | -0.30906 | 10.547 |
| geo_bl | 0.47946 | 2.5494 | 21.615 | 8.7844 | -7.4387 | 6.7423 | 0.31404 | -4.574 | -15.375 | -3.9842 |
| geo_bb | 0.86962 | 1.6406 | -1.5518 | -0.55957 | 18.51 | -0.5966 | 12.487 | -20.779 | 5.4764 | -5.6547 |
| geo_cbrc | -2.084 | -1.9389 | 4.915 | -7.3713 | 4.3895 | -6.5513 | -0.6289 | 7.8143 | -1.2736 | -1.313 |
| geo_lefr | 9.5592 | 4.627 | -6.17 | -1.8824 | -10.63 | 8.3376 | 7.0866 | 10.065 | 6.8377 | 1.2075 |
| geo_isprc | 2.6547 | -0.57625 | -18.431 | 15.918 | 0.70244 | -0.8006 | -10.044 | 5.2787 | 3.0461 | -5.6767 |
